# Examining the Influence of Social Network Factors on Weight Loss Among Latina and Non-Hispanic White Breast Cancer Survivors: Observational Cohort Study

**DOI:** 10.2196/77823

**Published:** 2026-05-07

**Authors:** Emily Janio, Michael A Hoyt, Kelly A Biegler, Sadeeka Al-Majid, Yunan Chen, Alfred Kobsa, Dara H Sorkin

**Affiliations:** 1 School of Medicine University of California, Irvine Irvine, CA United States; 2 Joe C. Wen School of Population & Public Health University of California, Irvine Irvine, CA United States; 3 School of Nursing California State University, Fullerton Fullerton, CA United States; 4 Donald Bren School of Information and Computer Sciences University of California, Irvine Irvine, CA United States

**Keywords:** Latina breast cancer survivors, mobile health app, self-discovery, health-related social support, health-related social control, undermining

## Abstract

**Background:**

Breast cancer is the most commonly diagnosed cancer among women and is the leading cause of cancer death among Latina individuals. Breast cancer survivors are at increased risk of obesity. Mobile health interventions have been shown to be an effective way of reducing the risk of weight gain. Less studied but also important is the extent to which social networks play a role in supporting or undermining weight loss efforts.

**Objective:**

We examined the association between 4 kinds of social network interactions and change in BMI among Latina and non-Hispanic White breast cancer survivors engaging in a mobile health app pilot study.

**Methods:**

Latina and non-Hispanic White breast cancer survivors were randomized to engage in either the *Mi Salud* or *Mi Vida, Mi Salud* app. *Mi Salud* allowed participants to engage in self-monitoring by recording their behaviors and symptoms. *Mi Vida, Mi Salud* used these same features in addition to a self-discovery feature that would summarize and report back this information to participants. We collected information on BMI and health-related social support; positive and negative health-related social control (which included persuasion and pressure, respectively); and undermining at baseline and after 12 weeks of the intervention.

**Results:**

While participants (non-Hispanic White n=22 and Latina n=22) in both study arms experienced decreased BMI over the 12-week period, this change in BMI did not differ according to ethnicity. Furthermore, change in social support was not associated with decreased BMI (B=−0.19, *P*=.12). However, the interaction between change in social support and ethnicity was significant, such that predicted margins were significant for non-Hispanic White individuals (B=−0.57, *P*=.02) but not for Latina individuals (B=−0.54, *P*=.72). Change in persuasion was not associated with change in BMI (B=0.072, *P*=.61); however, increased pressure was associated with increased BMI (B=0.66, *P*=.02). Finally, change in undermining was not associated with change in BMI (B=0.32, *P*=.11).

**Conclusions:**

Latina and non-Hispanic White participants did not differ in weight loss. However, our findings regarding social network involvement and change in BMI show the importance of considering social network processes in weight loss among breast cancer survivors. These findings buttress existing research suggesting the benefits of social support, particularly within specific cultural frameworks, while attempts to increase participants’ healthy behaviors that involve criticism can be detrimental to change efforts. Future research that builds on these findings is needed to elucidate the specific social network processes that may drive health behavior among diverse breast cancer survivors.

## Introduction

Breast cancer is the leading cause of cancer-related deaths among Latina individuals [1]. Latina breast cancer survivors also have higher rates of obesity relative to non-Hispanic White survivors [2,3], and less than 15% [4] adhere to nutrition and physical activity guidelines shown to aid in weight management [5,6]. Despite this, Latina individuals are underrepresented in oncology clinical trials [7,8], and most lifestyle interventions involving breast cancer survivors have focused on non-Hispanic White participants [9-11].

There has been steady growth in the development and testing of mobile apps as a means to deliver evidence-based interventions in cancer survivorship [12,13], including the development of mobile apps for Latina breast cancer survivors [14-16]. The widespread ownership of mobile devices [17] bolsters the possibility of enhanced reach of behavioral interventions. Despite their widespread popularity, few mobile cancer interventions are evidence-based [18-20]. This study focused on women participating in *Mi Vida, Mi Salud,* a culturally tailored mobile health intervention to promote weight loss among Latina breast cancer survivors through the use of *self-monitoring* and *self-discovery* (Sorkin, DH, unpublished data, October 2019). Self-monitoring is the process of collecting information [21]. Self-discovery can involve compiling this information for the user to identify and learn about trends in behavior and health outcomes [22]. While a prior study showed that engaging in self-monitoring or self-discovery did not have differential effects on BMI, as women in both groups lost a significant amount of weight over the study period (Sorkin, DH, unpublished data, October 2019), there may be other factors that influence health behaviors, including the social network involvement of meaningful others [23,24].

Network members can promote healthy behaviors by engaging in social support, which can be characterized by provisions of both emotional (eg, encouragement) or tangible (eg, rides to the gym) assistance [25,26]. When someone’s unhealthy behavioral choices put them at risk, those in their close social circle may attempt to encourage behavioral changes. This practice of seeking to effect or regulate the health behaviors of others is referred to as health-related social control [27]. The literature suggests that the effects of health-related social control attempts may depend, in part, on the types of strategies used by social network members. Efforts to prompt or persuade another person to improve the recipient’s health behaviors, termed persuasive social control, have been found to elicit positive health behavior change and positive emotional responses in some studies [28-30] but not others [31,32], whereas efforts that involve pressure, such as criticizing or expressing doubts about the recipient’s health behaviors, have often been found to be ineffective or even counterproductive in changing behavior [32]. Finally, the actions of individuals within a social network, whether deliberate or not, can sometimes hinder a person’s efforts to adopt healthier behaviors. Unlike health-related social support and social control, which aim to support or guide behavior, undermining refers to attitudes or actions that disrupt or obstruct progress toward key objectives, such as maintaining a healthy eating plan [33,34].

While there is extensive research on how social support affects health-related behaviors, the concepts of health-related social control and undermining have not been explored as thoroughly. Furthermore, the influence of these social processes on health-related outcomes of Latina breast cancer survivors has not been well-characterized [35]. Social network involvement may be particularly important to Latina individuals, as Hispanic individuals are more likely to rely on their social networks for health-related support relative to other ethnic groups [36]. A prior study of Latina breast cancer survivors found that social support for healthy eating from family members was high, but that family was also reported as a barrier to healthy eating, in part because participants felt they were unable to cook healthier foods as their family members would not enjoy them [35]. These findings suggest that the role that meaningful others play in influencing health behavior change is complex.

We expand on this prior work by assessing 3 conceptually distinct facets of social network members’ health-related involvement that might be expected to influence weight loss in a sample of Latina and non-Hispanic White breast cancer survivors participating in a behavioral weight loss pilot study. We expected that increases in health-related social support and the positive form of health-related social control (ie, persuasion) and decreases in the corrosive form of social control (ie, pressure) and undermining would be associated with weight loss.

## Methods

Methods of our study are detailed elsewhere (*under review*), and we describe them here in brief, in accordance with CONSORT-EHEALTH (Consolidated Standards of Reporting Trials of Electronic and Mobile Health Applications and Online Telehealth) reporting guidelines, where appropriate (Multimedia Appendix 1).

### Participant Recruitment

**Figure 1:**
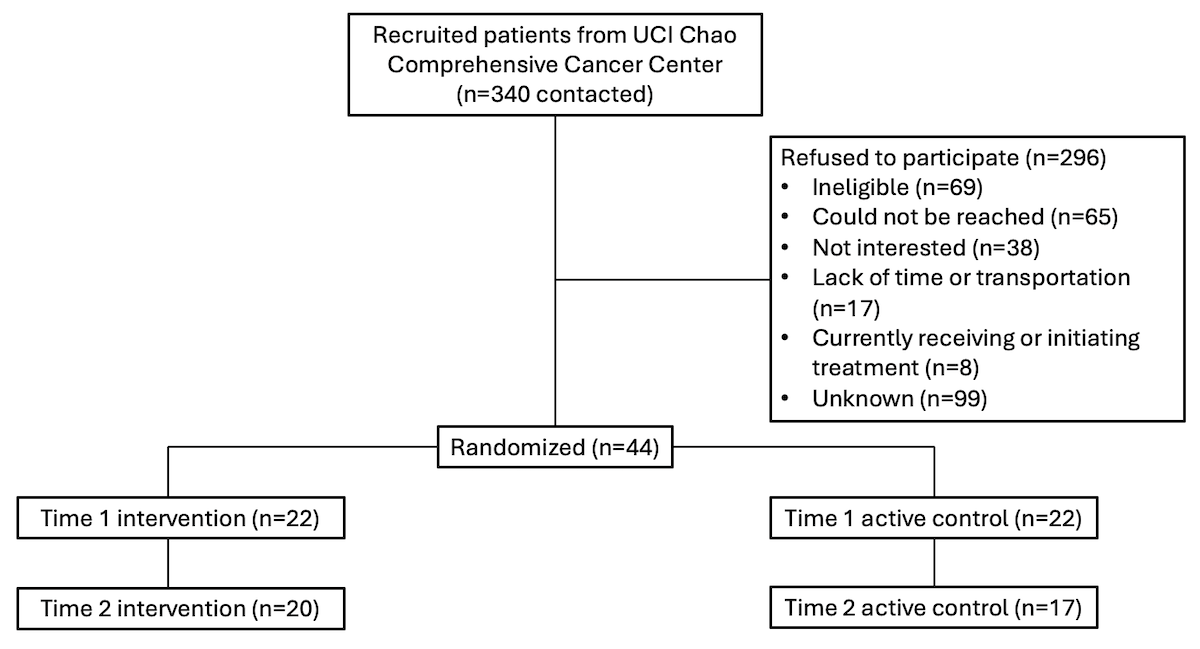
CONSORT-EHEALTH (Consolidated Standards of Reporting Trials of Electronic and Mobile Health Applications and Online Telehealth) diagram showing the recruitment and randomization of non-Hispanic White and Latina breast cancer survivors.

Participants were recruited from a large academic medical center in Southern California that draws patients including those residing in areas with some of the highest proportion of Latino individuals in the United States. Using data available in patient medical records, potential participants were identified as (1) female, (2) aged at least 18 years, (3) diagnosed with stage 1 to 3 breast cancer, (4) having completed active treatment, (5) having a BMI greater than or equal to 25 kg/m^2^ and less than or equal to 43 kg/m^2^ at study entry, and (6) self-identifying as either White and Hispanic or Latino and/or Spanish speaking. We excluded individuals for whom moderate physical activity was contraindicated and those with metastatic disease, recurrent cancer, visual or hearing impairment, or any major psychiatric and/or life-threatening illness that precluded their ability to consent. Potential participants were randomly selected to receive a letter from their health care provider describing the study and were contacted by telephone by study personnel. Through our institution’s National Cancer Institute–designated cancer center, we identified 3261 (n=617, 18.9% Latina) women who had been diagnosed with breast cancer and completed active treatment. A total of 275 women were contacted and 44 (16%) ultimately consented to participate in the study. The CONSORT (Consolidated Standards of Reporting Trials) diagram of our recruitment process is shown in Figure 1.

### Ethical Considerations

Study procedures were approved by the Institutional Review Board at the University of California, Irvine (2014-1543). All participants provided informed consent. Data were deidentified. Participants were compensated for participating in the study.

### Procedures

After providing consent, participants were stratified by ethnicity and randomized into an intervention or active comparison group: either *Mi Vida, Mi Salud* or *Mi Salud*, respectively. This was done via a form of adaptive randomization, minimization (1:1 ratio), because this was a small study and simple randomization could result in covariate imbalances [37]. The characteristics used for group assignment were age, time since diagnosis, stage of disease, whether patients had chemotherapy and/or radiation, and current use of aromatase inhibitors or tamoxifen [38]. All participants were given a smartphone preloaded with the corresponding mobile app. In *Mi Salud,* women recorded diet, exercise, fatigue, mood, sleep, and pain. *Mi Vida, Mi Salud* contained these same features as well as tools for self-discovery. The self-discovery feature presented the participant’s own data back to them through a series of filters. For example, participants were able to filter information pertaining to their diet, exercise, weight, or sleep by their mood or pain levels. An example of this is shown in Multimedia Appendix 2: in this image, word clouds show the association between what a participant has eaten and their symptoms, with red indicating fatigue and green indicating energy. The greater the size of the font, the more that food had been consumed. Therefore, the app allowed participants to assess the association between their behaviors and symptoms. Participants used the apps for 12 weeks from 2017 to 2018.

The apps were culturally and individually tailored, as they were available in both Spanish and English, and participants could record their decisions about food and exercise choices. For example, participants could record their mood and then rate its intensity on a scale.

### Measurements

At 3 time points (baseline, 6 weeks, and 12 weeks), we collected biometric and survey data from participants; however, we only focused on data collected at baseline and 12 weeks. We measured participants’ weight and height to calculate their BMI.

Surveys included items assessing health-related social support, health-related social control (eg, persuasion and pressure), and undermining [39]. Three items were used to assess health-related social support [32,40]. A sample item included, “Over the past month, how often did the important people in your life show appreciation for your efforts to stay on track with your diet or exercise regimen?” (0=not at all; 5=every day). The composite measure of health-related social support demonstrated good reliability in this sample at baseline (Cronbach α=0.90) and 12 weeks (Cronbach α=0.87) [32,40].

Seven items, 3 to assess persuasion and 4 to assess pressure, were derived from prior research to assess health-related social control [32,40]. Participants were asked to indicate the frequency with which their social network members sought to exercise health-related social control in the past month. A sample item for persuasion included, “Over the past month, how often did the important people in your life try to do something to get you to improve your food choices or exercise regimen?” A sample item for pressure included, “Over the past month, how often did the important people in your life restrict you from making poor food choices or from being physically inactive?” Responses were made on a 6-point scale (0=not at all; 5=every day), and composite variables were created to represent each strategy of health-related social control. Items pertaining to pressure and persuasion at baseline (Cronbach a=0.87 and Cronbach a=0.87, respectively) and at 12 weeks (Cronbach a=0.68 and Cronbach a=0.85, respectively) also had good reliability.

Health-related undermining was assessed with 7 items from the sabotage subscale of the Family and Friends Support for Heart Healthy Eating Habits scale [39]. Sample items included “refused to eat the healthy food you prepared” or “offered you high-sugar, high-fat foods.” Responses were made on a 6-point scale (0=never; 5=always). The composite measure demonstrated good reliability in this sample (Cronbach α for undermining at baseline and 12 weeks were α=0.79 and α=0.79, respectively).

At time 1, 22 non-Hispanic White and 22 Latina participants provided complete survey data. At time 2, of the 22 participants in each group, 19 non-Hispanic White (86.4%) and 18 (81.8%) Latina women provided complete data at the 12-week follow-up. Non-Hispanic White women used the mobile app more often than did Latina women, although this was marginal (non-Hispanic White: 60.5% of days vs Latina: 37.7% of days; *P*=.09); however, mobile app use data were available from only 18 (81.8%) Latina and 13 (68.4%) non-Hispanic White participants, indicating that more Latina participants actually downloaded and used the app compared with non-Hispanic White participants.

### Data Analysis

We used Fisher exact test and 2-tailed *t* tests to compare differences in demographics, changes in BMI, and changes in social network processes among Latina and non-Hispanic White participants, from baseline to 12-week follow-up. We then used linear regression to assess the association between group (1=*Mi Salud, Mi Vida* and 0=*Mi Salud* only) and BMI change score. We then used 4 separate linear regression models to assess the association between change in BMI as the outcome and change in social network process as the predictor. We first controlled for group, ethnicity (1=Latina, 0=non-Hispanic White), BMI at time 1, and social network process score at time 1. We then added terms showing the interaction between ethnicity and the change in the social network process. Models with significant interaction terms were then further probed by calculating the predictive margins of change in BMI according to change in social network processes among Latina and non-Hispanic White participants separately. Regression models used full information maximum likelihood estimation to address missing data at the 12-week follow-up. All analyses were conducted in Stata (version 16; StataCorp LLC) [41]. We reported significance at *P*≤.05 and a trending association at *P*≤.10.

## Results

Demographics are shown in Table 1. Non-Hispanic White participants had higher levels of education than Latina participants. Overall, the entire sample did lose weight over the 12-week period, with non-Hispanic White women as a group experiencing a greater decrease in BMI on average (Table 1). However, linear regression models showed that group was not significantly associated with a change in BMI (B=0.22, *P*=.54), as has been reported previously (*under review*). As noted in Table 2, Latina participants experienced a significant decrease in pressure (*P*<.001) and undermining (*P*=.02) over the course of the study.

Table 3 includes the results from adjusted models showing the association between change in social network process and change in BMI. Increased health-related social support was not associated with a change in BMI (B=−0.19; *P*=.12) but became significant with the addition of the interaction term (B=−0.57; *P*=.005), such that increased social support was associated with weight loss. Change in persuasion was not associated with change in BMI (B=0.072, *P*=.61). However, increased pressure was associated with weight gain (B=0.66, *P*=.02). Finally, change in undermining was not associated with change in BMI (B=0.32, *P*=.11).

**Figure 2:**
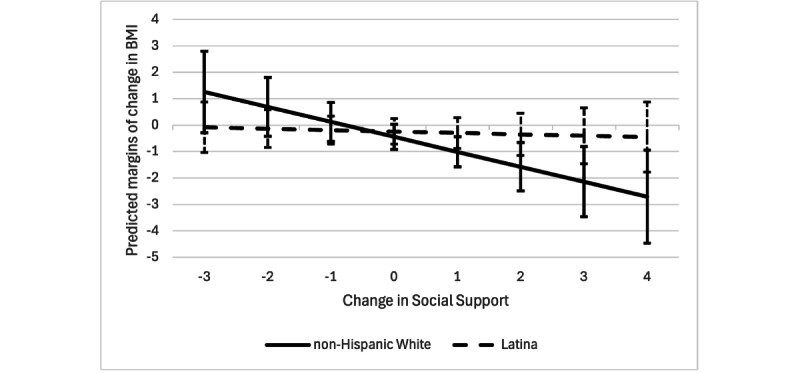
Predictive margins of change in BMI (y axis) for Latina and non-Hispanic White breast cancer survivors according to level of change in social support (x axis; n=37).

The interaction between change in social support and ethnicity was significant. Predicted margins were significant for non-Hispanic White participants (B=−0.57, *P*=.02) but not for Latina participants (B=−0.054, *P*=.72). The predicted margins of change in BMI for Latina and non-Hispanic White participants are presented in Figure 2.

**Table 1:** Demographic and health characteristics among Latina (n=22) and non-Hispanic White (n=22) breast cancer survivors participating in a mobile health app pilot study.

		Latina participants	Non-Hispanic White participants	*P* value	
**Marital status, n (%)**					.18
	Married	15 (68.2)	16 (72.7)		
	Divorced	3 (13.6)	6 (27.3)		
	Single	3 (13.6)	0 (0)		
	Other	1 (4.6)	0 (0)		
**Level of education, n (%)**					.001
	Grade School	5 (22.7)	0 (0)		
	High School	8 (36.4)	2 (9.1)		
	College	7 (31.8)	9 (40.9)		
	Graduate or professional	2 (9.1)	11 (50)		
**Employment status, n (%)**					.17
	Full-time	5 (22.7)	11 (50)		
	Part-time	7 (31.8)	5 (22.7)		
	Retired	2 (9.1)	4 (18.2)		
	Homemaker	4 (18.2)	1 (4.6)		
	Unemployed	4 (18.2)	1 (4.6)		
Years since diagnosis, mean (SD)		9.18 (5.06)	8.25 (4.77)	.53	
Age (years), mean (SD)		50.95 (7.93)	52.91 (10.84)	.50	
Proportion of days app used, mean (SD)		37.72 (33.90)	60.51 (36.89)	.09	
Change in BMI, mean (SD)		−0.15 (0.98)	−0.67 (1.07)	.12	
Time 1 BMI, mean (SD)		30.80 (4.37)	30.31 (3.88)	.69	
Time 2 BMI, mean (SD)		31.25 (4.53)	29.79 (4.17)	.31	

**Table 2:** Differences in average scores on social network involvement scales between time 1 (baseline) and time 2 (12 weeks) for non-Hispanic White and Latina breast cancer survivors.

	Latina participants				Non-Hispanic White participants		
	Time 1, mean (SD)	Time 2, mean (SD)	*P* value	Time 1, mean (SD)		Time 2, mean (SD)	*P* value
Social support	2.56 (1.85)	2.30 (1.60)	.56	1.81 (1.57)		2.11 (1.54)	.23
Persuasion	2.35 (1.67)	1.81 (1.67)	.20	1.18 (1.33)		0.96 (0.96)	.42
Pressure	2.08 (1.56)	0.60 (0.82)	<.001	0.39 (0.78)		0.25 (0.48)	.16
Undermining	1.77 (1.01)	1.02 (0.86)	.02	1.35 (0.84)		1.17 (0.90)	.31

**Table 3:** Linear regression analysis of models predicting change in BMI with and without interaction terms (n=44) among non-Hispanic White and Latina breast cancer survivors.

			Social support		Persuasion		Pressure		Undermining	
			B	*P* value	B	*P* value	B	*P* value	B	*P* value
**Model 1 (without interaction)**										
**Social Network Interaction Change**			−0.19	.12	0.072	.61	0.66	.02	0.32	.11
	**Ethnicity (non-Hispanic White)**									
		Latina	0.27	.4	0.24	.5	0.27	.47	0.71	.03
	**Time 1**									
		BMI	0.039	.33	0.013	.74	0.0028	.94	0.008	.83
		Social Network Interaction	0.12	.29	0.24	.08	0.66	.004	−0.00023	.99
	**Group (control)**									
		Intervention	0.28	.37	0.26	.43	0.17	.57	0.0097	.98
**Model 2 (with interaction)**										
**Social Network Interaction Change**			−0.57	.005	0.19	.41	0.72	.25	0.32	.11
	**Ethnicity (non-Hispanic White)**									
		Latina	0.2	.5	0.17	.64	0.25	.54	0.65	.11
	**Time 1**									
		BMI	0.032	.39	0.013	.75	0.0033	.93	0.0098	.8
		Social Network Interaction	0.12	.25	0.25	.06	0.67	.004	−0.0033	.99
	**Group (control)**									
		Intervention	0.24	.41	0.27	.4	0.17	.58	0.0097	.98
	**Interaction**									
		Ethnicity×Social Network Interaction	0.51	.02	−0.16	.52	−0.059	.91	−0.039	.82

## Discussion

### Principal Findings

This study assessed the association between social network involvement and change in BMI among Latina and non-Hispanic White breast cancer survivors in the context of a mobile health weight loss intervention. While not the primary focus of this manuscript, we note that there was not an association between change in BMI and participation in the intervention (*under review*), which mirrors the findings of other similar studies [42,43]. This may be an indication that inclusion of self-discovery in the intervention group was not substantially different from the functions provided within the active control group. Nevertheless, our entire sample experienced weight loss over the course of the 12 weeks.

Our findings build upon prior work assessing the association between different social network processes and health-related outcomes. There was no main effect of change in health-related social support on weight loss in the entire sample; however, increased health-related social support was associated with decreased BMI, particularly among non-Hispanic White women, highlighting a pattern of findings consistent with the literature [44]. The lack of association between social support and weight loss among Latinas was surprising given that prior research has shown a significant increase in health-related social support among Latina women with type 2 diabetes engaged in a weight loss intervention [23,45]. The degree to which social support is experienced may vary as a function of perceived illness burden. For example, some evidence suggests that Latina breast cancer survivors in particular express concerns about burdening their families [46], and perhaps this reluctance to seek or receive social support makes this type of network provision less effective for Latina women [46].

Regarding health-related social control, we found that persuasion was not associated with change in BMI. Although multiple studies have found this relationship, this pattern aligns with other work showing that persuasion is not always effective in promoting behavior change [23,31]. In contrast, pressure was associated with weight gain, which is consistent with prior work showing the negative effects of pressure in decreasing adherence to health behaviors [32,47,48]. Our study is among the first to demonstrate an association between increased pressure and weight gain.

Finally, we found that undermining was not associated with change in weight. Prior work has shown that the effects of undermining may vary according to health behaviors: in one study, undermining of physical activity was not associated with weight change, while undermining of healthy eating was associated with weight gain [48]. Future studies are needed to examine how undermining influences weight change among breast cancer survivors.

### Limitations

This pilot study has limitations. As this study was designed to obtain preliminary evidence regarding the feasibility of the *Mi Vida* intervention, it did not include more extensive follow-up assessments, thus limiting a more detailed analysis of the potentially reciprocal process of interpersonal involvement with weight loss efforts. A second limitation was the lack of consideration of individual goal setting. Prior work has shown that monitoring progress toward a set goal is an important aspect of attaining that goal [49]. Although all women were asked to lose between 1 and 2 lb (0.45 to 0.91 kg) per week, allowing for individual autonomy in determining a personal weight loss goal was likely to improve weight loss outcomes and also provide a clear goal that could be communicated to meaningful others. A third limitation of our study was that the measures of social network involvement did not identify the specific sources of the health-related social support, control, and undermining. Future research is needed to use finer-grained measures to evaluate how changes in the behavior of specific social network members over time relate to health outcomes.

### Conclusions

Nevertheless, this pilot study is among the first to report changes in social network interactions within the context of a weight loss intervention among breast cancer survivors. The inclusion of a large sample of Latina women is notable, given their increased risk for symptom burden and comorbidities, coupled with the lack of focus on examining social network involvement to increase healthy behaviors in this population. These findings can be used to inform future larger randomized controlled trials aimed at improving health behaviors among Latina breast cancer survivors.

## Appendix

Multimedia Appendix 1 CONSORT-eHEALTH checklist (V 1.6.1).

Multimedia Appendix 2 Example of the Mi Vida, Mi Salud interface.

## Abbreviations

CONSORT: Consolidated Standards of Reporting Trials
CONSORT-EHEALTH: Consolidated Standards of Reporting Trials of Electronic and Mobile Health Applications and Online Telehealth

## Data Availability

The datasets generated or analyzed during this study are available from the corresponding author on reasonable request.
