# Supplementary figures and images for: Examining the Influence of Social Network Factors on Weight Loss Among Latina and Non-Hispanic White Breast Cancer Survivors: Observational Cohort Study
Source: JMIR Form Res. 2026 May 7;10:e77823. doi: 10.2196/77823 (PMC13195378; doi:10.2196/77823)

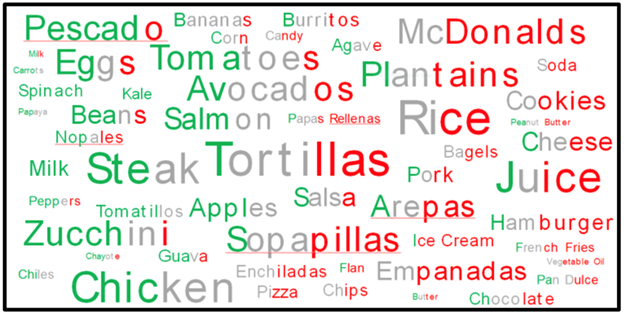

Supplement: Multimedia Appendix 2 [file formative_v10i1e77823_app2.png]
